# Extruder Path Analysis in Fused Deposition Modeling Using Thermal Imaging

**DOI:** 10.3390/polym17243310

**Published:** 2025-12-15

**Authors:** Juan M. Cañero-Nieto, Rafael J. Campo-Campo, Idanis B. Díaz-Bolaño, José F. Solano-Martos, Diego Vergara, Edwan A. Ariza-Echeverri, Crispulo E. Deluque-Toro

**Affiliations:** 1 Dept. Civil, Materials and Manufacturing Engineering, Escuela de Ingenierías Industriales, Universidad de Málaga, Andalucía Tech, Campus de Teatinos, 29071 Málaga, Spain; jmcanero@uma.es (J.M.C.-N.); jsolano@uma.es (J.F.S.-M.); 2 Grupo de Nuevos Materiales y Didáctica de las Ciencias, Facultad de Ingeniería, Universidad del Magdalena, Santa Marta 470004, Colombia; rafaelcampoj@unimagdalena.edu.co (R.J.C.-C.); cdeluque@unimagdalena.edu.co (C.E.D.-T.); 3 Grupo de Investigación y Desarrollo en Sistemas y Computación, Facultad de Ingeniería, Universidad del Magdalena, Santa Marta 470004, Colombia; idiaz@unimagdalena.edu.co; 4 Technology, Instruction and Design in Engineering and Education Research Group (TiDEE.rg), Catholic University of Avila, C/Canteros s/n, 05005 Ávila, Spain

**Keywords:** infrared thermography (IRT), process monitoring, in situ quality control, extruder path analysis, polylactic acid (PLA), additive manufacturing (AM), G-code

## Abstract

Fused deposition modeling (FDM) is one of the most widely adopted additive manufacturing (AM) technologies due to its accessibility and versatility; however, ensuring process reliability and product quality remains a significant challenge. This work introduces a novel methodology to evaluate the fidelity of programmed extruder head trajectories and speeds against those executed during the printing process. The approach integrates infrared thermography and image processing. A type-V ASTM D638-14 polylactic acid (PLA) specimen was fabricated using 16 layers, and its G-code data were systematically compared with kinematic variables extracted from long-wave infrared (LWIR) thermal images. The results demonstrate that the approach enables the detection of deviations in nozzle movement, providing valuable insights into layer deposition accuracy and serving as an early indicator for potential defect formation. This thermal image–based monitoring can serve as a non-invasive tool for in situ quality control (QC) in FDM, supporting process optimization and improved reliability of AM polymer components. These findings contribute to the advancement of smart sensing strategies for integration into industrial additive manufacturing workflows.

## 1. Introduction

Additive manufacturing (AM) enables the creation of three-dimensional objects by joining materials layer by layer [1] and has found extensive application in fields ranging from aerospace and automotive to biomedical [2]. Among the available materials, polymers remain the most widely utilized due to their properties, process simplicity, and low manufacturing costs [3]. Despite the technology’s rapid evolution since its inception [4] and its numerous advantages—such as mass customization, on-demand production, and waste reduction [5]—significant challenges persist. The primary challenge hindering the industrial implementation of AM is the variability and uncertainty in the structural properties of manufactured parts, which include microstructural heterogeneities and dispersed defects [1,4,6].

The final part quality is intrinsically dependent on a complex interplay of process parameters, such as material, build orientation, layer thickness, and infill pattern [3,7]. Deviations in these parameters can generate defects like cracks, porosity, residual stresses, and high surface roughness, all of which negatively impact the mechanical properties and service life of the component [6,8]. To address this, non-destructive testing (NDT) methods are employed, with non-contact techniques like thermography being of particular interest as they do not compromise the component’s integrity [8].

In the specific context of fused deposition modeling (FDM), a material extrusion (MEX) process, quality monitoring is divided into two approaches: printer health monitoring (assessing machine status via sensors) and product defect detection during printing (typically using visual cameras) [9]. In the latter domain, infrared thermography (IRT) has emerged as a powerful tool. IRT allows for non-contact, real-time temperature measurements, providing deeper insight into thermal processes, which is crucial in manufacturing supervision and materials science [10,11,12,13,14,15,16,17,18,19,20,21].

The recent literature has applied IR thermography to the FDM process to address various problems. One set of investigations has focused on defect detection and the characterization of thermal anomalies. For example, in situ monitoring has been used to compare cooling histories and detect anomalous thermal profiles [22] and for the detection of embedded defects in polylactic acid (PLA) specimens [23]. Others have analyzed raster temperature distribution for defect detection using machine learning models (SVM and KNN) [24,25] or have used IR sensors for real-time detection of voids, over-extrusion, and nozzle clogging based on deviations in melt pool temperature [26].

A second group of studies has focused on process characterization and model validation. These works have linked acquired temperature values with the 3D geometric model of the part [27], analyzed the inter-layer weld formation process (inter-layer strength) by evaluating cooling profiles [28], and investigated the overall thermal evolution of ABS parts to improve mechanical properties [29]. Similarly, long-wave infrared (LWIR) thermography has been used to compare experimental temperature profiles with numerical models [30] and to investigate the cooling of the extrudate at the nozzle outlet, identifying radial temperature gradients that negatively affect process quality [31]. A notable study by Chen et al. [32] proposed a methodology to capture and compensate for the geometrical accuracy of PLA parts by processing thermal images, estimating the error in the deposition path to correct it in real-time.

While these studies [22,23,24,25,26,27,28,29,30,31,32] demonstrate thermography’s ability to monitor thermal properties and detect resulting defects, a fundamental gap remains: the direct validation of the extruder’s kinematic fidelity. That is, the quantification of how closely the actual nozzle path and speed follow the programmed paths and speeds in the G-code. Most research, including [32], focuses on correcting the resulting part geometry rather than diagnosing the source of the deviation (the tool’s motion) that causes the error.

Therefore, the main objective of this work is to propose a novel methodology that closes this gap by integrating LWIR thermography with G-code data analysis to evaluate the extruder path conformity in FDM. This methodology establishes a geometric calibration procedure for the thermal camera, enabling the precise measurement of nozzle position and speed using pattern matching techniques. It then systematically compares the estimated kinematics (from thermal images) with the programmed paths (from G-code) during the printing of a PLA specimen. Finally, new statistical indices are proposed to quantify the degree of manufacturing compliance, establishing a robust foundation for early defect detection and process optimization.

## 2. Experimental Methodology

The methodology in this study involved a dual-workflow analysis of an FDM printing process. First, the G-code file, generated by a slicer from the specimen’s CAD model, was processed to extract the nominal extruder head trajectories and speeds. Second, thermal images were acquired concurrently during the printing process. These images were then processed using computer vision algorithms to estimate the actual kinematic variables (position and speed) of the extruder head. Finally, the nominal data (from G-code) and the estimated data (from thermal images) were systematically compared on a layer-by-layer basis using statistical metrics to quantify process fidelity and detect deviations. The experiment involves printing a single specimen of a specific geometry where the process parameters are fixed during the printing setup.

### 2.1. Materials and Test Specimen

**Figure 1:**
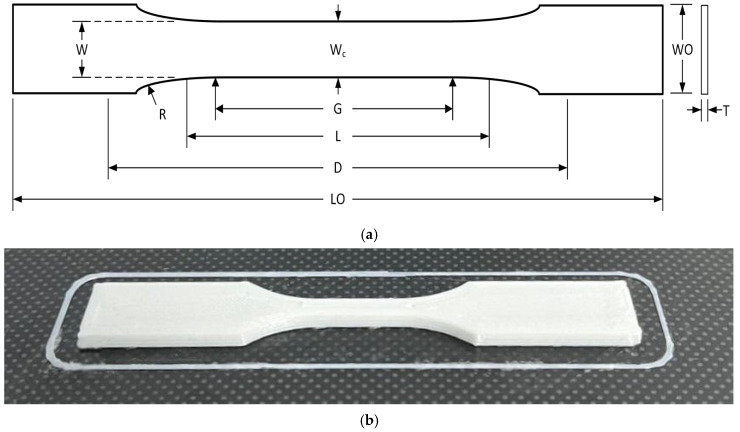
(**a**) Dimensional drawing of tensile specimen by ASTM D638-14 and (**b**) a printed PLA type-V specimen.

The feedstock material used was a commercial polylactic acid (PLA) filament with a 1.75 mm diameter (±0.05 mm tolerance) and a density of 1.24 g/cm^3^. A type-V specimen (Figure 1), as defined by the ASTM D638-14 “Standard Test Method for Tensile Properties of Plastics” [33], was selected for the experimental procedure. Table 1 details the nominal dimensions and tolerances for this specimen.

### 2.2. FDM Printer and Slicing Parameters

The specimens were fabricated using a Creality Ender-3 V2 printer (Shenzhen Creality 3D Technology Co., Ltd., Shenzhen, China) equipped with a 0.4 mm extrusion nozzle. All slicing operations and G-code generation were performed using PrusaSlicer software (version 2.6.0). The primary thermal parameters were set and maintained at 55 °C for the build platform and 212 °C for the extrusion nozzle. A flat build orientation was used, and the specimen was constructed from 16 layers, each with a 0.2 mm height.

**Figure 2:**
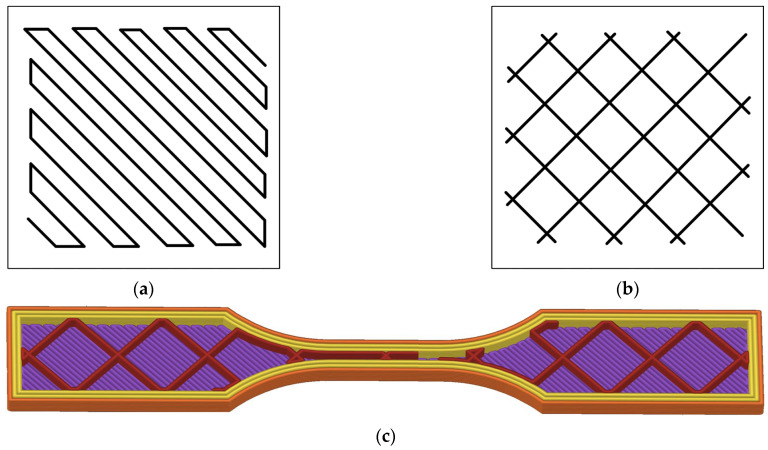
(**a**) Monotonic infill pattern, (**b**) grid infill pattern, and (**c**) 3D internal view of the specimen.

**Figure 3:**
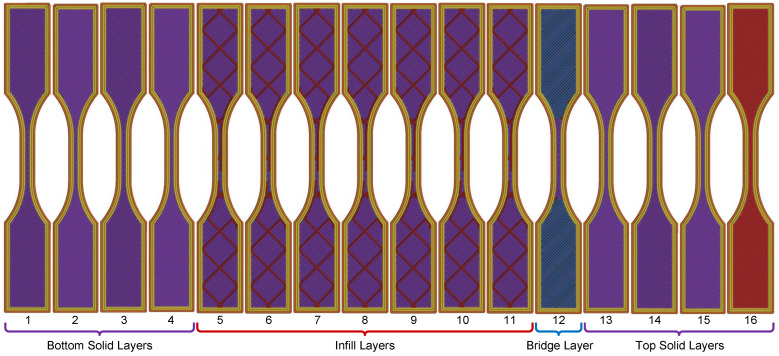
Identification layer number and layer types that comprise the specimen.

The specimen’s internal architecture was defined by distinct infill patterns (Figure 2). A monotonic pattern was selected for the solid top and bottom layers (Figure 2a), while a 15% density grid pattern was used for the infill sections (Figure 2b). The 16-layer build sequence was functionally grouped by type, as illustrated in Figure 3: four bottom solid layers (Layers 1–4), seven grid infill layers (Layers 5–11), one bridging layer (Layer 12), and four top solid layers (Layers 13–16). A comprehensive list of all printing parameters is summarized in Table 2.

### 2.3. Thermal Imaging System and Software

A long-wave infrared (LWIR) camera (Optris Xi 400 from Optris GmbH & Co., Berlin, Germany) was used for thermal monitoring. The camera featured an uncooled FPA detector (382 × 288 pixels), a 17 μm square pitch, a 7.5 to 13 μm spectral range, and a thermal sensitivity of 80 mK. It was equipped with a 20 mm focal length lens. For this study, the camera was configured to measure temperatures from 0 °C to 250 °C and was set to a 27 Hz frame rate. Image acquisition was handled via a USB connection, running a custom software application. This application integrated the Optris [34] library for image acquisition and the Halcon MVTec [35] library for image processing.

**Figure 4:**
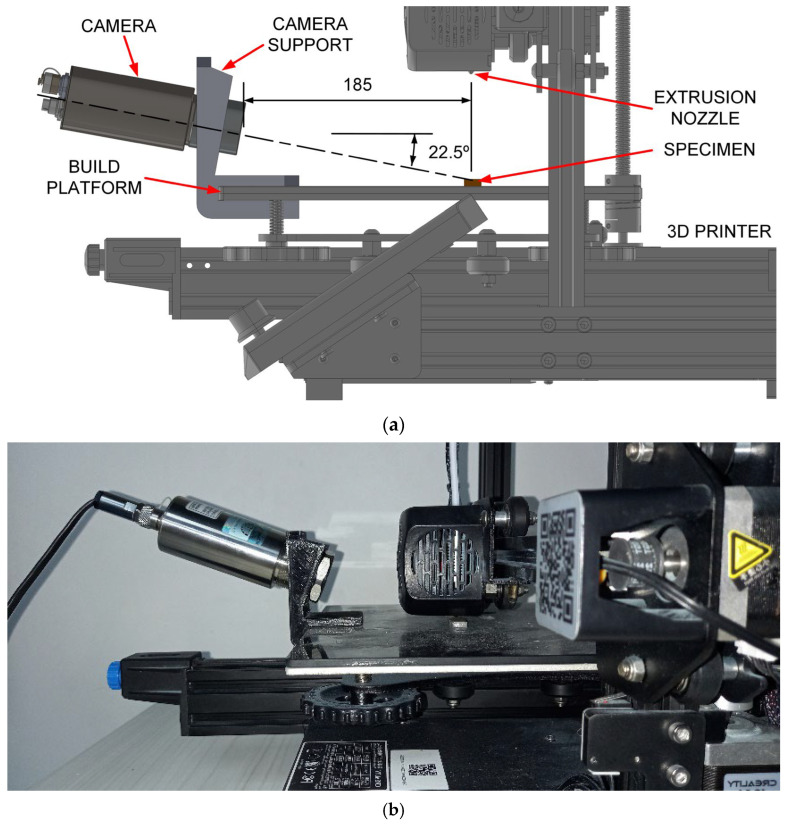
Test bench: (**a**) lateral view of main elements and (**b**) camera and camera support fixed to the printer build platform.

The experimental setup is illustrated in Figure 4. The LWIR camera was secured to the printer’s build platform using a custom support (Figure 4b). As shown in the schematic (Figure 4a), the camera was positioned at a horizontal distance of approximately 185 mm from the specimen’s center and tilted at a 22.5-degree downward angle relative to the platform. This positioning resulted in a field of view (FOV) of approximately 69 mm and an instantaneous field of view (IFOV) of 0.18 mm at the specimen’s surface.

### 2.4. Data Processing Workflow

**Figure 5:**
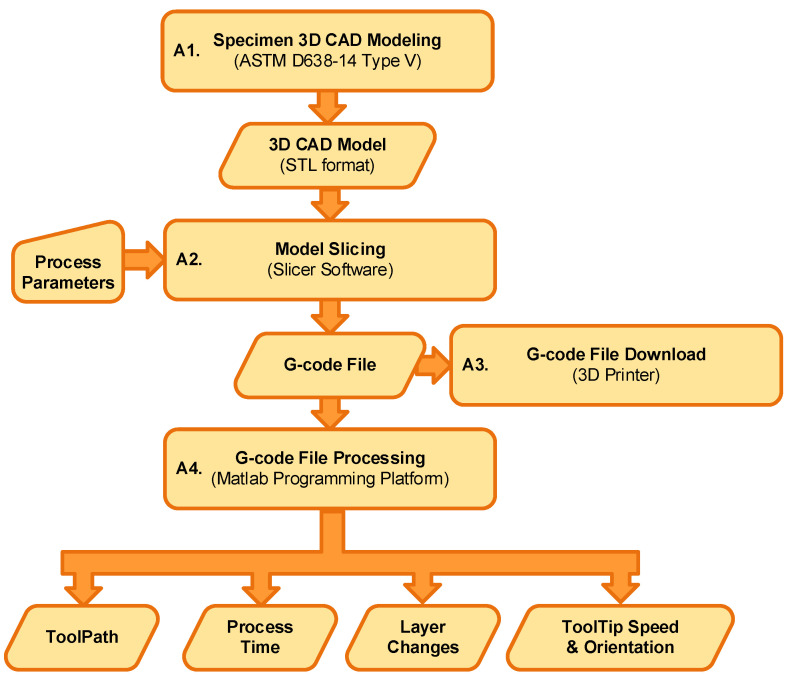
G-code processing workflow.

**Figure 6:**
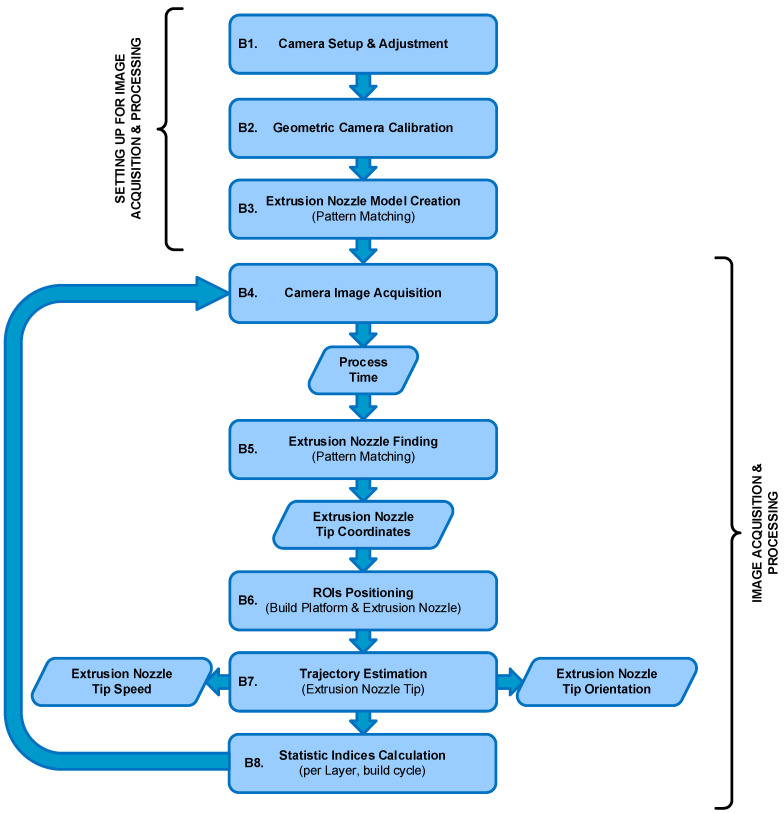
Vision-based workflow.

Data from the printing process were acquired and processed through two parallel workflows (Figure 5 and Figure 6). The first workflow involved processing the G-code file to extract the nominal process parameters, primarily the extruder’s toolpath and speed (Figure 5). The second, vision-based workflow, involved acquiring and processing thermal images to estimate the actual extruder kinematics (position and speed) during the build (Figure 6).

Following data acquisition, the outputs of both workflows were systematically compared to quantify the process fidelity. This comparative analysis used graphical plots to show the evolution of key variables and employed standard error metrics, such as the Mean Absolute Error (MAE) and the Root Mean Square Error (RMSE), to measure deviations.

#### 2.4.1. The G-Code Processing Workflow

**Figure 7:**
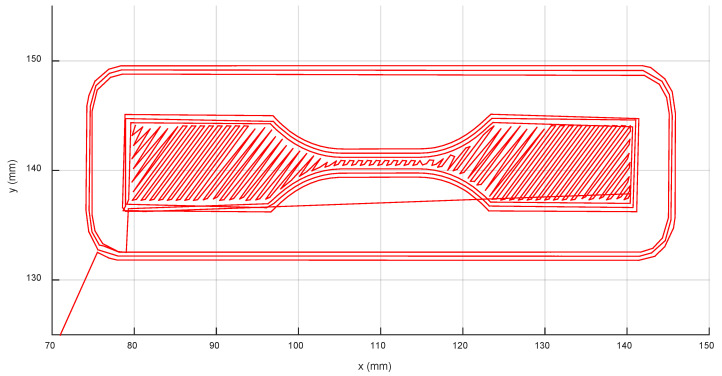
Layer toolpath in the XY plane processed from the G-code.

The G-code processing workflow (Figure 5) began with the specimen’s 3D CAD modeling (A1). The model was created, and the corresponding STL (Standard Tessellation Language) file was exported using Solidworks 2018 software. Next (A2), the G-code file was generated by importing the STL file into the PrusaSlicer tool and applying the process parameters detailed in Table 2. After the G-code file was loaded onto the 3D printer (A3), a parallel copy was processed (A4) using a custom script in the MATLAB programming platform. The goal of this processing was to parse the file and extract the nominal values for key process variables, including the extruder tooltip position (X, Y, Z), speed (feed rate), toolpath orientation (Figure 7), cumulative elapsed time, and all layer change events.

**Figure 8:**
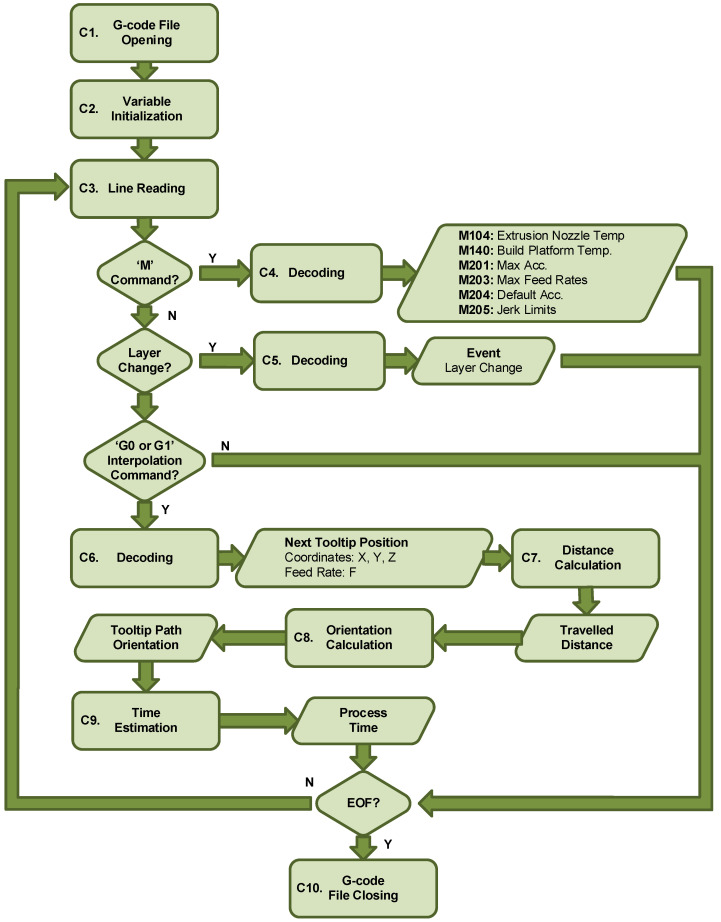
Matlab G-code processing algorithm.

**Figure 9:**
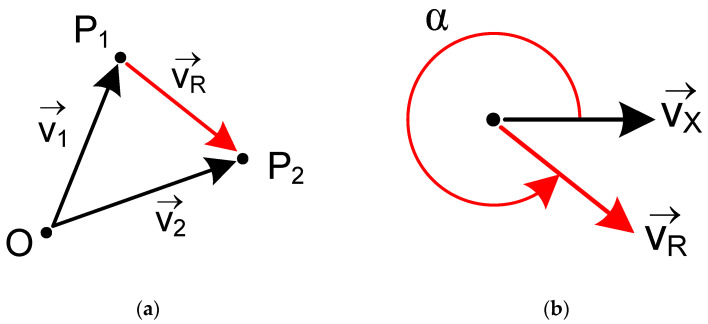
(**a**) Vector resulting from displacement between two points and (**b**) angle relative to the X-axis.

Figure 8 shows the flowchart for the G-code file processing algorithm. Once the file was opened (C1), the algorithm iteratively read each line (C3) and processed it by decoding its content until the end-of-file (EOF) mark was detected, at which point the file was closed (C11). The algorithm first determined the type of information contained in the line: a comment, a miscellaneous (M) command, or a linear motion (G) command. If a miscellaneous command was found, it was decoded (C4) to obtain temperature settings for the nozzle and bed (M104, M140), acceleration and feed rate limits (M201, M203), default acceleration (M204), and jerk speed limits (M205). If the line contained a comment, it was processed (C5) to identify layer change events. Finally (C6), if the line contained a G0 or G1 linear interpolation command, the target 3D coordinates (X, Y, Z) and the motion feed rate (F) were obtained. From the coordinates of the start ($P_{1}$) and target ($P_{2}$) points, the travel distance was calculated (C7) as the Euclidean norm of the resultant vector $\vec{V_{R}}$ (Figure 9a). This vector was then used to calculate the toolpath orientation angle, α, relative to the X-axis (C8), expressed in degrees from 0° to 360° (Figure 9b). Equation (1) shows the calculation of α from the arctangent of the ratio of the cross-product’s norm and the dot product of the vectors.

(1) $$\alpha =\tan^{-1}\frac{\left\|\vec{V_{X}}\times \vec{V_{R}}\right\|}{\vec{V_{X}}\cdot \vec{V_{R}}}$$

**Figure 10:**
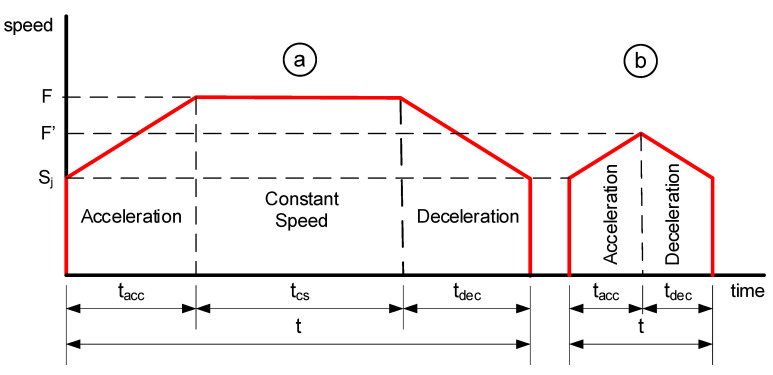
Speed profiles: (**a**) Trapezoidal and (**b**) triangular.

The estimation (C9) of the elapsed time, *t*, for the tooltip to travel a linear distance depended on the speed profile type (Figure 10). If the programmed feed rate, F, was reached and maintained, the speed profile was trapezoidal (Figure 10a); if it was not reached, it was triangular (Figure 10b). Thus, time t was the sum of the elapsed times during the acceleration ($t_{acc}$), constant speed ($t_{cs}$), and deceleration ($t_{dec}$) stages, with $t_{cs}=0$ for the triangular profile. This calculation also considered the jerk speed, $S_{J}$: the maximum speed at which the printer could change direction between consecutive displacements. For a trapezoidal profile (Figure 10a), the total distance, d, was calculated as:

(2) $$d={2\cdot d}_{acc}+\;d_{cs}$$

where $d_{acc}$ and $d_{cs}$ are the distances traveled during the acceleration (or deceleration) and constant speed profile stages. These values can be obtained by integrating the area under each profile stage.

(3) $$d_{acc}=t_{acc}\cdot \left[\frac{\left(F-S_{J}\right)}{2}+S_{J}\right]$$

(4) $$d_{cs}=t_{cs}\cdot F$$

The time $t_{acc}$ can be obtained from the known values of F, $S_{j}$, and A read from the G-code file.

(5) $$t_{acc}=\frac{\left(F-S_{J}\right)}{A}$$

Rearranging Equations (2) and (4), the value of $t_{cs}$ can be calculated as:

(6) $$t_{cs}=\frac{d_{cs}}{F}=\frac{d-2\cdot d_{acc}}{F}$$

In the case of the triangular speed profile (Figure 10b), the total distance can be obtained from Equation (2) where $d_{cs}=0$. This means that the total distance traveled only depends on $d_{acc}$, which can be calculated again by integrating the area under the speed profile.

(7) $$d_{acc}=t_{acc}\cdot \left[S_{j}+\frac{F^{\prime }-S_{j}}{2}\right]$$

The value of $t_{acc}$ can be defined by Equation (5) where now the feed value takes the unknown value $F^{\prime }$. Solving $F^{\prime }$ from Equation (5) and substituting it into (7), the total distance can be written as:

(8) $$d=2\cdot t_{acc}\cdot \left[S_{j}+\frac{A\cdot t_{acc}}{2}\right]$$

Rearranging Equation (8), the $t_{acc}$ value can be calculated by solving the second-grade equation.

(9) $$A\cdot t_{acc}^{2}+2\cdot S_{j}\cdot t_{acc}-d=0$$

Finally, the elapsed time t, estimated for traveling between two points, was added to the time counter that determines the total time elapsed since the start of the printing process.

To assess the consistency of the thermal monitoring methodology, the analysis exploited the repetitive nature of the layer-by-layer deposition process. Although a single specimen was printed, the G-code programming generated multiple layers with identical toolpaths and process parameters, effectively creating internal experimental repetitions.

The layers were grouped for analysis as follows:

- Group A (Shells): Layers 2, 4, 13, and 15, which share identical perimeter paths and nominal speeds.
- Group B (Infill): Layers 5 through 11, which share identical infill patterns and densities.
- Unique Layers: Layers 1 (first layer), 12, and 16 (bridge) possess unique pathing and were excluded from the repeatability comparison.

#### 2.4.2. Vision-Based Workflow and Camera Calibration

**Figure 11:**
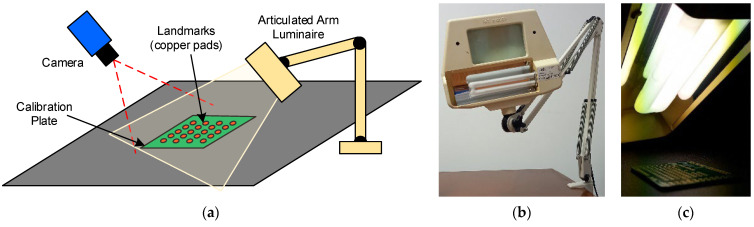
(**a**) Camera geometric calibration setup, (**b**) articulated arm luminaire, and (**c**) heat-radiated calibration plate.

**Figure 12:**
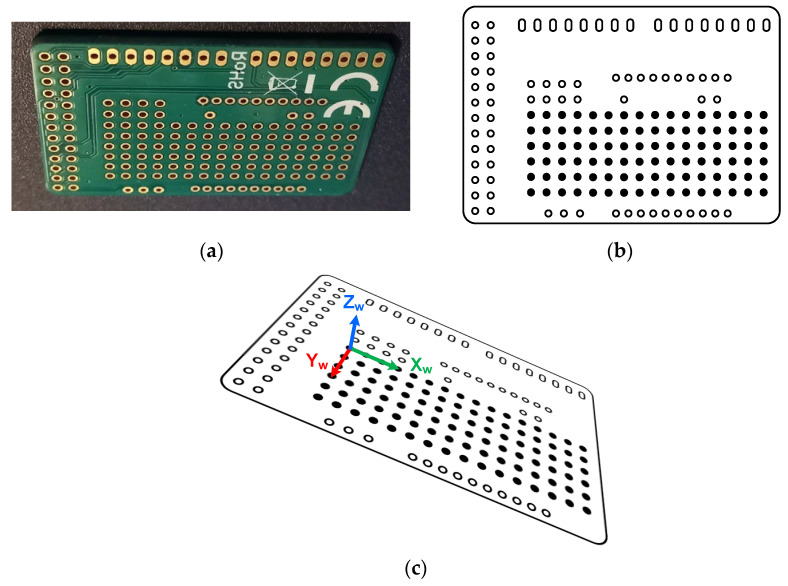
(**a**) Calibration plate, (**b**) landmark points (black filled), and (**c**) 3D world coordinates system.

The vision-based workflow (Figure 6) involved monitoring the specimen by acquiring and processing thermal images during the production run. This was composed of preliminary system setup steps followed by cyclical image acquisition and processing tasks. In the first stage (B1), initial adjustments were made for both the camera and the subsequent image processing. Specifically, the camera parameters and its position were configured as described in Section 2.3 and Section 2.4. Emissivity values were also set; a value of 0.75 was used for the build surface (based on direct measurements), and 0.92 was used for the PLA [36]. The determination of the extrusion nozzle emissivity value is discussed in a later section. Camera geometric calibration (B2) was a fundamental step for accurately retrieving geometrical information from the thermal images. Its main objective was the determination of the camera’s intrinsic and extrinsic parameters [37]. The intrinsic parameters are characteristic of the camera (e.g., internal orientation, focal length, lens distortion), while the extrinsic parameters define the transformation between the 3D world scene and the 2D camera coordinate system [38]. The calibration method (Figure 11a) consisted of acquiring thermal images of a calibration plate placed in various positions and orientations relative to the camera. The plate contained a series of known 3D points (landmarks). A fluorescent lamp (Figure 11b,c) was used to create thermal contrast. The high contrast between the emissivity of the copper pads (ε ≈ 0.03) and the fiberglass board surface (ε ≈ 0.75) caused the heat radiation to reflect differently, making the landmarks detectable in the thermal images and enabling geometric calibration. The calibration plate used was an FR-4 type printed circuit board (PCB) (1.6 mm × 51 mm × 35 mm) featuring drilled holes, each surrounded by a copper-plated annular ring (pad) (Figure 12a). Only the central 16 × 6 matrix of landmarks, with a 2.54 mm (1 inch) pitch, was used for calibration (Figure 12b). The division model [39] was chosen over the polynomial model [40] for calculating the intrinsic parameters, as it uses only one parameter to model distortion compared to the five required by the polynomial model.

**Figure 13:**
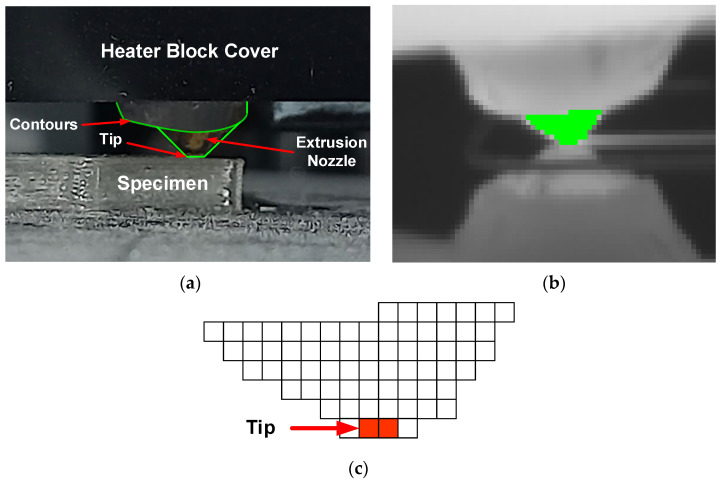
(**a**) Contours extracted from extrusion nozzle zone, (**b**) extrusion nozzle mask, and (**c**) tip position in mask.

The gray-level thermal image processing to obtain the 2D position of the landmarks began by applying a Sobel filter [41] to find the landmarks’ contours. Subsequently, thresholding and morphological operations were performed on these contours to find the 2D coordinates of the center of each landmark. Finally, a correspondence was determined between the extracted 2D landmark coordinates and their known 3D world coordinates (Xw, Yw, Zw in Figure 12c) to compute the camera’s intrinsic and extrinsic parameters. After calibration, a shape model of the extrusion nozzle was built (B3) based on its extracted contours (Figure 13a). This model was later used (B5) to find the nozzle’s position in each acquired image by applying pattern matching techniques [42,43,44]. The cyclical processing tasks (Figure 6) began by acquiring a new 16-bit grayscale image from the camera (B4). The raw data stream for each image contained a timestamp, which was used for cumulative time tracking. Then (B5), the extrusion nozzle was located within the image using the pattern matching algorithm to find its position coordinates. A region of interest (ROI) was created based on these coordinates and processed using adaptive image thresholding [45] to generate a binary mask overlapping the nozzle (Figure 13b).

From this mask, the nozzle tip’s position was calculated (midpoint of the bottom row, Figure 13c) and transformed into the 3D world coordinate system using the previously determined extrinsic parameters. This transformation enabled the accurate measurement of toolpath variables (B7). The nozzle’s speed (B7) was estimated from the Euclidean distance traveled by the tip between the current and previous images and the elapsed time between their respective timestamps. The orientation relative to the X-axis, α, was also estimated using Equation (1). Finally (B8), basic statistical indices (median, standard deviation, min/max) were calculated per layer and for the total build cycle to support the subsequent comparative analysis.

## 3. Results and Discussion

### 3.1. Validation of the Vision-Based Workflow: Camera Calibration

**Figure 14:**
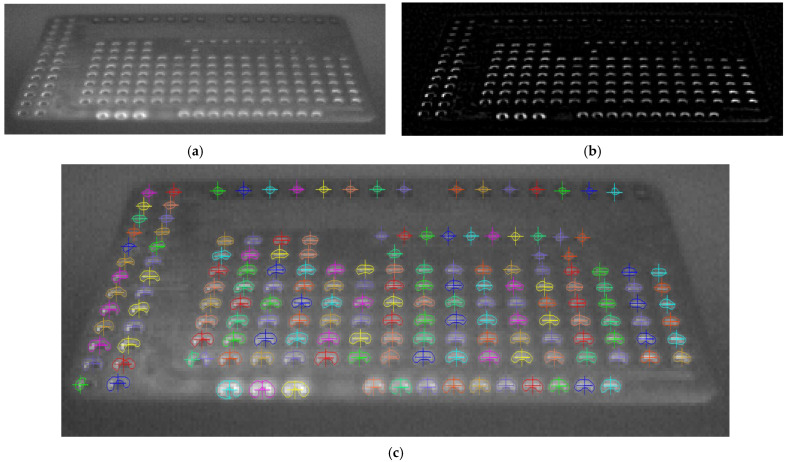
(**a**) Gray-level thermal image, (**b**) contours extraction with Sobel filter, and (**c**) landmarks found (color overlayed).

The foundational step for the vision-based workflow was the geometric calibration of the LWIR camera. The primary result of this stage is the successful validation of the image processing pipeline used to detect the calibration landmarks, which is presented in Figure 14. The original grayscale thermal image (Figure 14a) demonstrates the challenge: the low thermal contrast between the copper pads (ε ≈ 0.03) and the fiberglass board (ε ≈ 0.75) provides poor visual definition. However, applying a Sobel filter (Figure 14b) effectively converted these subtle thermal gradients into high-contrast edges. This edge detection was the critical step that enabled robust processing, allowing for subsequent thresholding and morphological operations to accurately identify and find the center of each landmark, as shown in Figure 14c.

**Figure 15:**
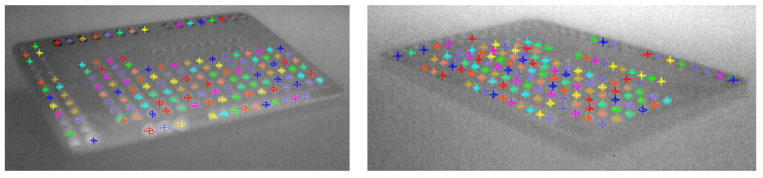
Calibration plate landmarks found (marked with colored crosses) in different positions and orientations.

This detection method proved to be highly robust, as it was successfully applied to all 12 calibration images, which captured the plate in various positions and orientations (Figure 15). The successful and repeatable detection of these landmarks across multiple perspectives was essential for calculating the camera’s intrinsic and extrinsic parameters.

The significance of this result is that it serves as the “ground truth” for the entire study. The calculated transformation parameters are what enabled the conversion of the extrusion nozzle’s 2D pixel coordinates (measured in subsequent thermal images) into a 3D real-world coordinate system (in millimeters). Without this accurate calibration, a direct and metrologically valid comparison between the estimated toolpath (from the camera) and the nominal toolpath (from the G-code) would be impossible.

To evaluate the quality of the thermal-camera calibration performed using the PCB plate, the Root Mean Square Error (RMSE) was used as the primary accuracy metric. The RMSE was computed as the square root of the average squared differences between the 2D image landmarks detected in the thermal frame and the corresponding reprojected 3D world points obtained from the calibration model. The resulting RMSE value of 0.8723 pixels indicates a satisfactory calibration accuracy for the camera resolution and lens configuration employed in this study, and is suitable for capturing the geometric information required for the kinematic analysis.

**Figure 16:**
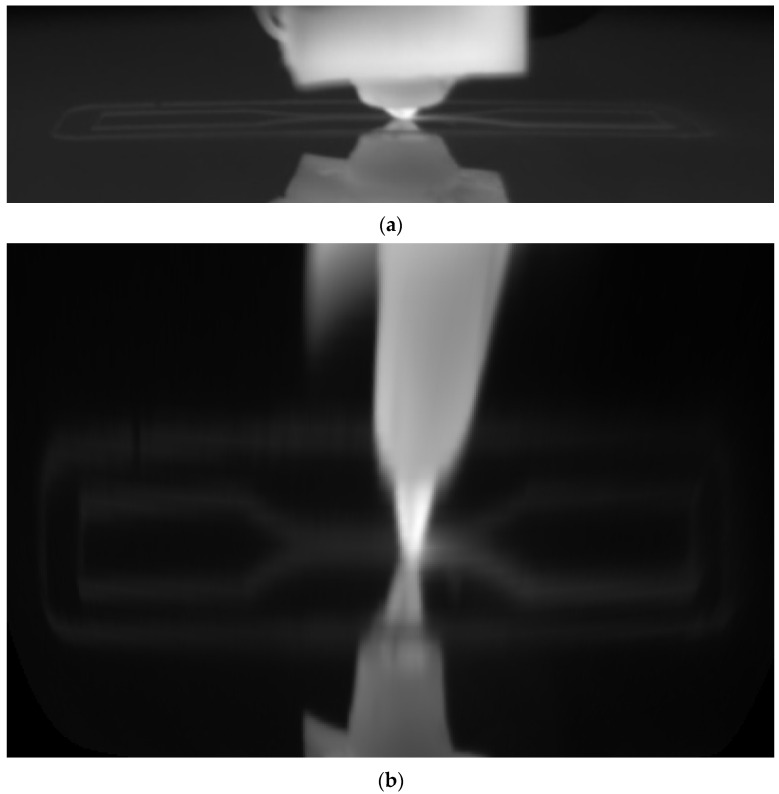
Raw (**a**) and transformed (**b**) image during the specimen printing.

A direct application and validation of the calculated calibration parameters are shown in Figure 16. Figure 16a presents the raw thermal image as captured by the camera. Due to the 22.5° mounting angle specified in the methodology, the image exhibits significant perspective distortion (or keystoning). In this raw view, the specimen appears angled, and measurements in pixels are not uniform; a 10-pixel movement at the bottom of the image (closer to the camera) does not correspond to the same real-world distance as a 10-pixel movement at the top.

Figure 16b demonstrates the power of the calibration: it shows the same image after applying the transformation matrix derived from the extrinsic and intrinsic parameters. The image has been mathematically rectified, removing both lens and perspective distortion. This process transforms the angled view into a flat, “bird’s-eye” (or top-down) view, aligning the image plane with the printer’s X-Y build plane.

The critical importance of this result is that it enables metrologically accurate measurements. In the rectified view (Figure 16b), pixel distances are now proportional to real-world distances (in millimeters) across the entire frame. This orthorectification is the essential prerequisite for accurately tracking the extruder nozzle’s position and speed, as any kinematic deviations measured in this view directly correspond to real-world deviations from the nominal G-code path.

### 3.2. Analysis of Extruder Kinematic Fidelity: Speed and Orientation

**Figure 17:**
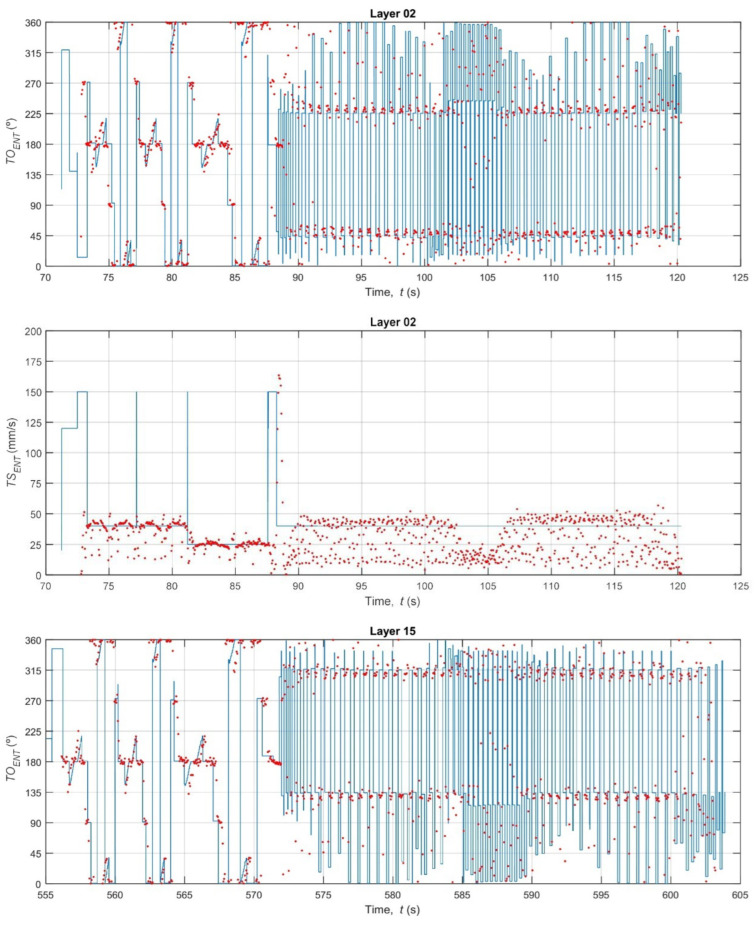

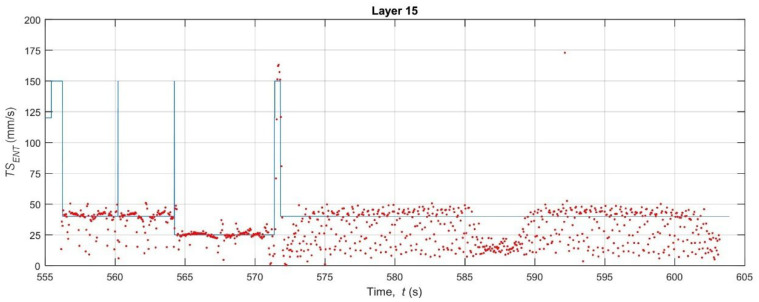
Bottom and top layers.

**Figure 18:**
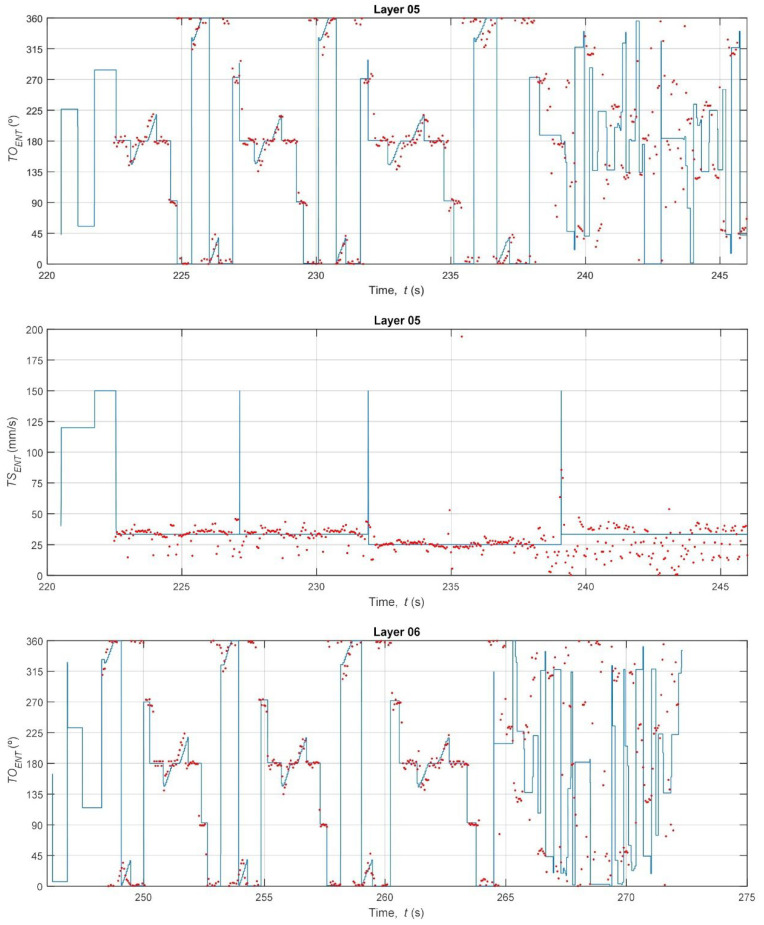

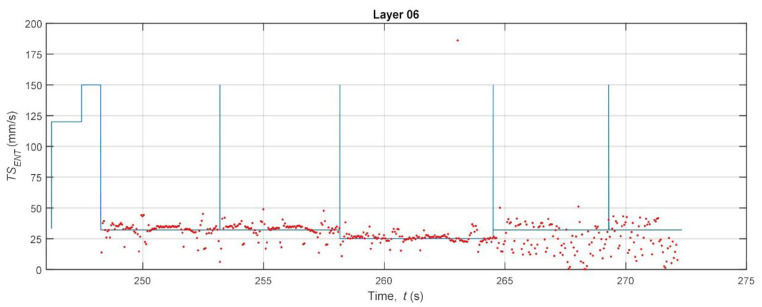
Grid infill pattern.

**Figure 19:**
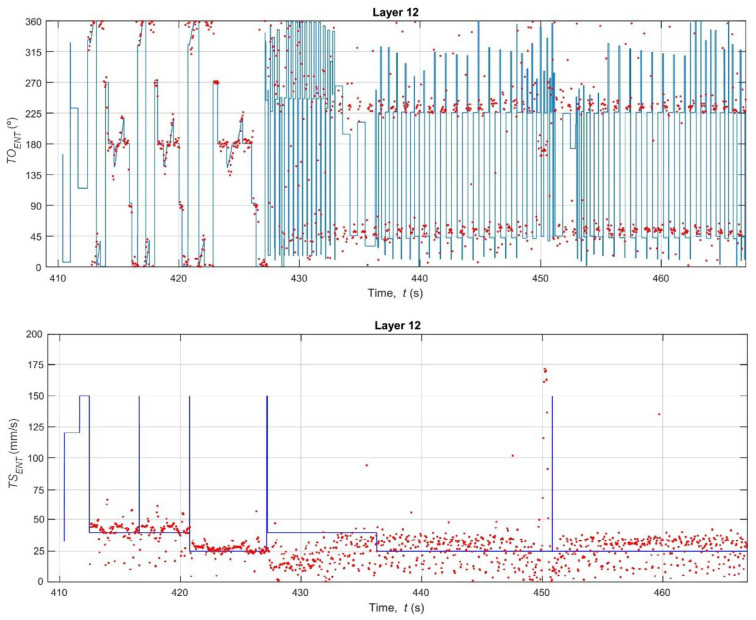
Bridge Layer.

**Figure 20:**
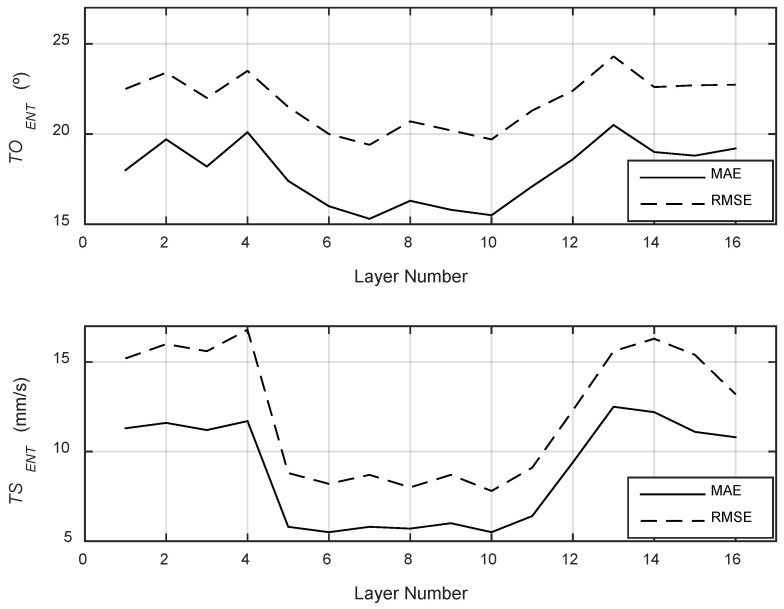
MAE and RMSE values for extrusion nozzle tip trajectory orientation and speed.

With the vision system calibrated, the core analysis of the study was performed: a high-fidelity, time-synchronous comparison between the nominal kinematics (derived from G-code) and the estimated kinematics (measured by the thermal camera). Figure 17, Figure 18 and Figure 19 provide qualitative, time-series visualizations of this comparison for different layer types, while Table 3 and Figure 20 present the quantitative statistical summary of the kinematic errors (MAE and RMSE) for the entire 16-layer build.

A visual inspection of the time-series plots reveals the fundamental difference between the idealized G-code commands and the real-world mechanical process. The nominal data (blue line) represents a perfect digital signal, characterized by instantaneous changes in speed (*TS_ENT_*) and orientation (*TO_ENT_*). For example, the *TS_ENT_* plot for Layer 02 (Figure 17) shows the speed instantly jumping from the base extrusion feed rate (≈30–40 mm/s) to the high-speed travel move (150 mm/s) and back. Conversely, the estimated data (red dots) from the thermal camera captures the noisy, analog nature of the physical world. The nozzle’s speed does not change instantly; it must accelerate and decelerate. This physical limitation is evident in the clusters of red dots, which lag behind the commanded G-code speeds.

A critical observation is the system’s inability to capture high-speed G0 travel moves. In the *TS_ENT_* plots (e.g., Figure 17, t = 88 s), the G-code commands a 150 mm/s travel speed, but the camera system (at 27 Hz) is too slow to capture this rapid movement. The nozzle has already departed and arrived at its destination before the next frame is acquired. This measurement limitation is a key source of error and explains the large, sporadic discrepancies in the speed data.

The quantitative analysis of the kinematic errors, summarized in Table 3 and plotted in Figure 20, provides the most significant insights. The data reveals that the fidelity of the printing process is not uniform but is directly correlated with the type of toolpath being executed (i.e., solid vs. infill layers).

The analysis of speed error (*TS_ENT_*) shows a distinct, repeating pattern. The Mean Absolute Error (MAE) for speed is significantly lower during the grid infill layers (Layers 5–11, MAE ≈ 5.5–6.4 mm/s) compared to the solid bottom layers (Layers 1–4, MAE ≈ 11.3–11.7 mm/s) and solid top/bridge layers (Layers 12–16, MAE ≈ 9.4–12.5 mm/s).

This clear distinction reveals a noteworthy difference. The 15% density grid infill (Layers 5–11) consists of short, repetitive, and relatively slow extrusion moves (see Figure 18). The printer spends the vast majority of its time at or near the programmed infill speed (~32 mm/s), which the camera can accurately track. In contrast, the solid layers and bridge layer (Figure 17 and Figure 19) involve long perimeter paths and, crucially, more frequent and longer high-speed G0 travel moves to reposition the nozzle across the specimen. As noted in the qualitative analysis, the camera system fails to measure these 150 mm/s travel moves, while the G-code logs them perfectly. This discrepancy, where the G-code reports 150 mm/s and the camera reports a much lower speed, creates large error spikes that significantly inflate the MAE and RMSE for all solid layers. This finding demonstrates that the proposed methodology is sensitive enough to differentiate between infill and solid toolpath strategies based on their kinematic error profiles.

The orientation error (*TO_ENT_*) reveals an alternative yet equally significant aspect of system behavior. As shown in Figure 20 (top) and Table 3, the orientation error is consistently high and noisy across all 16 layers (MAE ≈ 15.3–20.5°, RMSE ≈ 19.4–24.3°). This high error is not a failure of the measurement system but rather a quantification of the printer’s physical dynamics. The G-code (blue line in Figure 17, Figure 18 and Figure 19) commands instantaneous 90° or 180° changes in direction. In reality, the physical extruder head must decelerate to 0 mm/s, change direction (a moment where its orientation is briefly undefined or stationary), and then accelerate in the new direction. The 27 Hz camera captures this physical lag and the subsequent stabilization, which the idealized G-code model completely ignores. The large gap between the MAE (average error) and the RMSE (which heavily penalizes large errors) confirms that this error is not constant but is driven by large, sporadic error spikes that occur precisely at these turning points. This result suggests that orientation fidelity is a highly sensitive metric for capturing the printer’s real-world dynamic limitations (jerk and acceleration), which are known sources of printing defects like ringing and corner bulging.

To verify the internal consistency of the monitoring methodology, a statistical analysis was performed based on the layer grouping strategy. For the Shell layers (Layers 2, 4, 13, and 15), where the toolpath and nominal speed (60 mm/s) were identical, the method showed high repeatability. The Mean Absolute Error (MAE) for the orientation (*TO_ENT_*) resulted in a mean of 19.8° with a standard deviation of just 0.73°. Similarly, the speed error (*TS_ENT_*) for these layers showed a mean MAE of 11.7 mm/s (σ = 0.58 mm/s). For the Infill layers (Layers 5–11), which featured a distinct zig-zag pattern, the method demonstrated even higher stability. The orientation error (*TO_ENT_*) averaged 16.2° (σ = 0.78°), while the speed error (*TS_ENT_*) dropped significantly to a mean of 5.8 mm/s with a very low standard deviation of 0.31 mm/s. These low standard deviations across grouped layers indicate that the proposed thermal analysis method is consistent and capable of distinguishing between different printing strategies (e.g., shell vs. infill) with high reproducibility.

The investigation was conducted for a single print. However, several of the 16 layers have an exact repetition of both the toolpath and the print speed values. Based on this, it is possible to group the identical layers and analyze the potential variability in speed and orientation variables.

Table 4 shows three distinct groups, each with the same nominal toolpath and speeds. Group I consists of top or bottom solid layers with a monotonic infill oriented at −45° (layers 2, 4, 13, and 15), Group II also consists of top or bottom solid layers with a monotonic infill at +45° (layers 3 and 14), and Group III consists of layers belonging to the grid infill pattern (layers 5, 6, 7, 8, 9, 10, and 11). Layers 1,12, and 16 (bridge layer) are not grouped because they are unique and distinct in their programming, and it makes no sense to compare them to the rest.

In each group, the average (Avg) and standard deviation (Std) values calculated from the MAE and RMSE values of the *TS_ENT_* and *TO_ENT_* variables of each layer (Table 3) are indicated.

Overall, there is narrow variability within each group in both the MAE and RMSE of *TS_ENT_* and *TO_ENT_*. This could indicate good reproducibility and consistency in error estimation when comparing layers with the same toolpath and speed. The average values of groups I and II are similar, which suggests that the inclination of the monotonic infill (±45°) has little effect on the results, so both groups could be merged. Again, a lower error in the feed rate is generally observed. It is important to note that the present work represents a foundational stage in a broader research effort aimed at linking extruder kinematic deviations with the dimensional accuracy, microstructural conformity, and mechanical performance of FDM-printed parts. At this stage, the methodology was intentionally focused on evaluating whether thermal image processing can reliably quantify deviations in extruder orientation and speed along the programmed toolpath. Establishing the feasibility, stability, and repeatability of this analysis is a necessary prerequisite before attempting to correlate these error profiles with physical or mechanical consequences in the printed specimens. Future work will build on the validated methodology presented here to investigate whether layers exhibiting higher MAE or RMSE values indeed correspond to localized geometric deviations, microstructural discontinuities, or reductions in mechanical properties.

The findings from this kinematic analysis confirm that the proposed thermal-vision methodology is a viable, non-invasive tool for in situ process monitoring. The system successfully moves beyond simple anomaly detection (i.e., “is the print failing?”) to a more profound process fidelity analysis (i.e., “is the printer behaving as programmed?”). The ability to distinguish the error profiles of different toolpath types (solid vs. infill) is a significant step toward creating dynamic, layer-specific quality metrics.

However, the analysis also highlights clear limitations that define the path for future work. The current 27 Hz frame rate and point-to-point speed calculation (based on the previous frame) are susceptible to aliasing and fail to capture high-frequency travel moves. A clear avenue for improvement would be to pair a higher frame rate camera with more advanced tracking algorithms. For instance, rather than simple point-to-point estimation, a regression-based approach (e.g., fitting a local polynomial curve or using a Kalman filter) to the detected nozzle positions over time would provide a much smoother, more accurate estimation of instantaneous velocity and acceleration, even in the presence of measurement noise.

Because this work represents an initial validation of the proposed methodology, the analyses were intentionally performed on a single printed specimen. Despite this limitation, a qualitative assessment of repeatability was carried out in Section 3.2 by grouping layers that shared identical toolpaths and programmed speeds. The resulting error profiles showed consistent patterns across layers of the same type. This coherence in both orientation- and speed-error behavior demonstrated that the methodology could capture reproducible kinematic deviations when printing conditions remain constant. These results support the feasibility and reliability of the proposed thermal-image-based approach and indicate its potential for expansion toward more comprehensive studies. Future work could incorporate multiple specimens and a full experimental design to enable statistically robust validation and to further investigate the relationship between kinematic fidelity, microstructural variations, and mechanical performance.

Furthermore, while the division model [39] used for geometric calibration proved sufficient for this proof-of-concept, future work aimed at sub-millimeter metrology could benefit from implementing a more complex polynomial distortion model [40]. Such a model could correct for more subtle, non-linear lens distortions, further improving the accuracy of the world-coordinate transformation.

This study served as a proof-of-concept, prioritizing the validation of the thermal image processing technique for detecting extruder orientation and speed conformity. With this approach validated, future investigations could implement a rigorous Design of Experiments (DoE) to systematically evaluate the impact of critical printing parameters. Specifically, variables such as material deposition rate, infill density, and scanning strategies might be analyzed to determine their individual and interactive effects on the thermal history and resulting quality of the printed parts.

It is also important to note that this study was conceived as a feasibility analysis of the proposed thermal-image-based methodology; therefore, all results were obtained from a single printed specimen. The purpose of this approach was to evaluate whether the method was capable of reliably estimating the extruder’s orientation and speed conformity along the programmed G-code path before conducting a broader experimental campaign. Although only one print was analyzed, subsequent grouping of layers sharing identical toolpath characteristics enabled a qualitative assessment of the repeatability of the observed error profiles. Layers printed with the same programmed speed and trajectory exhibited highly similar patterns in both orientation and speed errors, suggesting a consistent behaviour of the extruder kinematics under identical printing conditions.

Finally, the greatest limitation of the current study is the single-camera setup, which restricts the analysis to a 2D plane and is susceptible to occlusions (e.g., the heater block or the nozzle itself obscuring the exact point of deposition). A significant advancement would be the implementation of a synchronized, stereoscopic system (i.e., two cameras). This would enable a full 3D reconstruction of the toolpath, eliminating occlusions. Such a setup would unlock the ultimate goal of in situ quality control: moving beyond tracking the nozzle to tracking the deposited bead itself. This would allow for the real-time measurement of critical geometric and thermal parameters of the actual part—such as bead width, height, and inter-layer cooling rate—linking process fidelity directly to final part quality.

## 4. Conclusions

This work successfully developed and validated a non-invasive, dual-workflow methodology for the in situ monitoring of the FDM process. By integrating a calibrated LWIR thermal camera with G-code parsing, the study moved beyond simple thermal monitoring to perform a high-fidelity kinematic process fidelity analysis, comparing the nominal (programmed) extruder toolpath against the estimated (actual) toolpath in real-time. The experiment involves printing a single specimen of a specific geometry (type-V ASTM D638-14) and material (PLA) with one set of process parameters.

The primary contributions of this study are twofold. First, the methodology proved to be a robust tool for quantifying kinematic fidelity, successfully validating the geometric calibration, image rectification, and nozzle tracking pipeline. Second, the analysis of the kinematic errors (MAE and RMSE) yielded significant insights into the printing process:

- The system is highly sensitive to the toolpath strategy, clearly distinguishing the kinematic error profiles of different layer types. The Mean Absolute Error (MAE) for speed was significantly lower during the 15% grid infill layers (MAE ≈ 5.5–6.4 mm/s) compared to the solid layers (MAE ≈ 9.4–12.5 mm/s). This discrepancy was found to be driven by the system’s 27 Hz frame rate, which was insufficient to capture the high-speed (150 mm/s) G0 travel moves prevalent in solid layer strategies.
- The orientation error (*TO_ENT_*) was consistently high across all layers (MAE ≈ 15.3–20.5°). This finding is not a measurement failure but rather the first-time quantification of the physical lag, jerk, and stabilization time the extruder head experiences during rapid directional changes—dynamics that are idealized and ignored by the nominal G-code. This metric serves as a direct measurement of the printer’s mechanical limitations.

These findings demonstrate that kinematic fidelity, particularly orientation error, can serve as a valuable new index for in situ quality control. While it is premature to draw a direct correlation to specific final part defects, the high orientation errors logically align with known defect mechanisms such as ringing (vibration) and corner bulging, which are caused by the exact acceleration and jerk limitations that this method successfully measured.

This study provides a validated methodology for quantifying extruder kinematic fidelity using thermal image processing. While the analysis successfully identifies variations in orientation and speed error along the toolpath, it does not yet evaluate whether layers with higher error magnitudes result in measurable dimensional deviations or mechanical deterioration. This limitation is intentional and reflects the preliminary nature of the study, whose primary purpose was to assess the feasibility and constraints of the proposed methodology. Having established a robust basis for detecting kinematic error patterns, future research will focus on correlating these deviations with microstructural analyses and mechanical testing to determine their practical implications on printed part quality.

This study highlights clear limitations and provides a roadmap for future work. The 27 Hz frame rate and point-to-point estimation proved insufficient for high-speed tracking. Future iterations should incorporate higher-speed cameras and more robust state-estimation algorithms (e.g., Kalman filters or regression models) to accurately model acceleration. Furthermore, the single-camera setup is limited to 2D and suffers from occlusion. A significant advancement would be the implementation of a synchronized, stereoscopic thermal camera system. Such a setup would not only enable full 3D toolpath reconstruction but would also unlock the ultimate goal of this research: shifting the monitoring focus from the nozzle to the deposited bead itself. By applying advanced artificial intelligence (AI) and machine learning models to the 3D geometry and thermal history of the bead, a direct link could be established between these in situ kinematic and thermal process deviations and the final mechanical and geometric quality of the printed part.

## Figures and Tables

**Table 1:** ASTM D638-14 type-V specimen dimensions.

Dimension	Description	Value (mm)	Tolerance (mm)
W	Width of narrow section	3.18	±0.5
L	Length of narrow section	9.53	±0.5
WO	Width overall, min	9.53	±3.18
LO	Length overall, min	63.5	no max
G	Gage length	7.62	±0.25
D	Distance between grips	25.4	±5
R	Radius of fillet	12.7	±1
T	Thickness	3.2	±0.4

**Table 2:** 3D printer settings.

Parameter	Value	Parameter	Value
Nozzle diameter	0.4 mm	Nozzle temperature	212 °C
Extrusion width	0.4 mm	Bed temperature	55 °C
Layer height	0.2 mm	Infill density	15%
Total layers	16	Grid pattern infill layers	7
Solid bottom layers	4	Solid top layers	4
Bottom/top infill rater angle	±45°	Bottom/top infill pattern	monotonic
Bridging layers	1	Bridging infill pattern	monotonic
Bridging infill raster angle	45°	Infill speed (Grid)	≈32 mm/s
Perimeter (per layer)	2 turns	External perimeter (per layer)	1 turn

**Table 3:** Summary of statistical results for motion analysis (per layer).

Layer	*N*		Time (s)			${TO}_{ENT}$ (°)			${TS}_{ENT}$ (mm/s)	
			$t_{start}$	$d_{layer}$		MAE	RMSE		MAE	RMSE
1	1413		0.0	72.0		18.0	22.5		11.3	15.2
2	951		72.1	47.9		19.7	23.4		11.6	16.0
3	945		120.1	50.1		18.2	22.0		11.2	15.6
4	950		170.2	49.3		20.1	23.5		11.7	16.8
5	588		219.5	26.6		17.4	21.5		5.8	8.8
6	478		246.2	26.3		16.0	20.0		5.5	8.2
7	483		272.5	25.2		15.3	19.4		5.8	8.7
8	480		297.7	25.8		16.3	20.7		5.7	8.0
9	482		323.5	25.6		15.8	20.2		6.0	8.7
10	443		349.1	25.7		15.5	19.7		5.5	7.8
11	438		374.8	26.3		17.1	21.3		6.4	9.1
12	1129		401.1	59.0		18.6	22.4		9.4	12.3
13	940		460.1	47.0		20.5	24.3		12.5	15.6
14	954		507.1	48.7		19.0	22.6		12.2	16.3
15	942		555.8	48.3		18.8	22.7		11.1	15.4
16	953		604.1	48.8		19.2	22.73		10.8	13.2

**Table 4:** Summary of statistical results for motion analysis (by group).

Layer Group	Layer Type		${TO}_{ENT}$ (°)					${TS}_{ENT}$			
			MAE		RMSE			MAE		RMSE	
			Avg	Std	Avg	Std		Avg	Std	Avg	Std
I	Top/Bottom (−45°)		19.8	0.73	23.5	0.66		11.7	0.58	16.0	0.62
II	Top/Bottom (+45°)		18.6	0.57	23.1	0.64		12.0	0.35	16.6	0.35
III	Infill (grid pattern)		16.2	0.79	20.4	0.80		5.8	0.31	8.5	0.48

## Data Availability

The original contributions presented in this study are included in the articl. Further inquiries can be directed to the corresponding author.
